# Use of patient reported outcome measures in daily clinical practice – A way to optimize treatment of patients?

**DOI:** 10.1111/aogs.14501

**Published:** 2023-03-11

**Authors:** Maria Monberg Feenstra, Kristian Kidholm, Martin Rudnicki

**Affiliations:** ^1^ Department of Obstetrics and Gynecology Odense University Hospital Odense Denmark; ^2^ Faculty of Health Science, Department of Clinical Research University of Southern Denmark Odense Denmark; ^3^ Centre of Innovative Medical Technology (CIMT) Odense University Hospital Odense Denmark; ^4^ Faculty of Health Science, Department of Clinical Research Odense University Hospital Odense Denmark

During recent decades, there has been a growing interest of using patient questionnaires for more than effect measurement in clinical practice. This includes screening, visitation, and the use of questionnaires as a dialogue‐ and decision support in the meeting between clinicians and patients. The interest is in both Denmark as well as international.

One example of patient questionnaires is Patient Reported Outcome Measures (PROM), which are questions measuring the patient's own perception of their health status.^1^ The use of PROM allows capturing the patient's own perspective on their disease, current severity, quality of life and self‐management of their disease. Thus, the aim is to measure all relevant aspects related to a patient's health status, as reported by the patient, without a clinician's influence.^2^

Previously, the use of PROM has generally included questionnaires in paper‐versions, but the development of digital tools enables the data to be distributed, collected and stored in a database as a supplement to the patient's electronic medical journal. This includes the possibility to send out questionnaires automatically to patients during treatment, however ensuring that the rules in relation to General Data Protection Regulation are respected. This may not always be easy but in the Region of Southern Denmark, this has been regulated by use of secure systems integrated in the electronic medical journal by which it is possible to send out questionnaires directly to patients by a regional secured application* (*App ‘My Hospital/Mit Sygehus’) and/or Web (www.mit.rsyd.dk) with a personal identifier (NemId/MitID). Further, this system allows the patients to answer PROM questionnaires by smartphone, tablet or computer, capturing data in a database, which are linked to the individual patient's medical journal.

The use of PROM has most frequently been allocated to chronic disease such as cancer, diabetes, chronic obstructive lung disease, inflammatory bowel disease, rheumatoid arthritis or ischemic heart disease but also in maternity care PROM have been used.^1^, ^3^ The explanation is the prevalence, symptom burden and the need for long‐term follow‐ups, and this may be done using PROM. However, in paper versions this is quite complicated and time consuming. The electronic capturing of PROM data may then be very useful by being able to create a quick and systematic overview of patients' responses and preferably supported by a severity‐algorithm. In addition, digital collection of PROM can often be done without costs for the health care institution or with only minor costs per patient.

A problem using questionnaires is whether these are validated, i.e., content validity. Often, PROM is used in specific populations without prior validation or even without validation in the specific group of interest. Reasons are many as validation of questionnaires can be time consuming, costly and optimally requires a multidisciplinary team.^4^

Recently, the use of PROM including patients with chronic diseases were reviewed to explore existing evidence and whether the use of PROM improved health, decreased outpatient clinic visit etc.^5^ The review disclosed that in many studies the outcomes were not described in details as well as the use of non‐ or validated questionnaires. Further, the purpose of the studies was not always clear. In general, studies did not demonstrate any specific effects and no safe conclusion could be made in relation to improvement of health, earlier discharge from hospitals etc. In one study following women with ischemic heart disease, even a higher mortality rate was observed. Thus, the use of PROM still needs to be evaluated and especially the aim behind the use needs to be very clear.

Similarly, Chen et al^3^ recently observed limitation in relation to the use of PROM in maternity care. The systematic review demonstrated limitations such as lack of generalizability and narrow scopes in investigating impact suggesting the need for widening the study population, including different types of PROMs and considering the effects of PRMs at different levels and domains of healthcare.

Further, a recent review described a catalogue of available PROM's in relation to endometriosis. The review was carried out since the use of PROM's are not used in routine clinical care and they may facilitate discussion of the relevant expectations to the effects of PROM and identification of issues that are not easily raised during consultations. In total 48 PROM's were identified including different aspect such as quality of life, painful symptoms, psychological effects of pain etc. However, the authors also indicate that clinicians are reluctant to introduce PROM as they might see it as an increased workload. The use of digital data, however, demands transfer of data immediately to the clinician allowing the use during consultation and thereby the use of digitalization.^6^

Currently, the clinicians have to be able to use more advanced digitalization of the medical records, other digital systems to get access to secure data, i.e., the use of digitalization of ultrasound scans, and at the same time be able to get access to patient data in previous records from earlier hospitalization. This is time‐consuming and many clinicians need two screens at their workstation in order to have an overview of all digital platforms. In addition, clinicians must further develop their digital‐ and communicative skills, when using new IT systems for collection of data on PROM and addressing patients' responses during routine visits. This may not be well accepted by clinicians unless new initiatives improve clinical outcomes, patient satisfaction or may be helpful in other areas.

During the last 3 years our department has focused on introduction of PROM in a digital version for patients with endometriosis attending our outpatient clinic. The aim was to improve visitation (allocation of outpatient visit to a doctor or a nurse, and consultation by relevant methods, such as phone or video consultations, physical consultation or no consultation), and to strengthen patient involvement. Taking the above‐mentioned criteria into account, the aim of the implementation of PROMs has to be clear, and at the same time it is important not to overestimate the effect of PROMs on clinical health outcomes.

Validation of the PROM focused on face‐ and content‐validity as well as reliability (test–retest) as a first‐step towards a full psychometric evaluation. Furthermore, an IT platform had to be created in order to send out questionnaires through a secure system, ensuring data to be captured by a database integrated partly into the patients' medical record. Finally, an algorithm was developed and tested to ensure optimal visitation and not to overlook any patient.

In conclusion, the value of the use of PROM still needs to be clarified – does it add substantial new and relevant information, and can it be implemented in a clinical setting without causing increased workload for the clinicians? Even though the use of digitalized PROMs may be complicated and costly, the implementation of PROM in the future in digital versions will enable safe information about the patients' perspectives used for visitation and follow‐up of chronic diseases etc., but the effect on clinical health outcomes and patient empowerment and clinicians' satisfaction of the use of PROMs need to be settled.

## References

[1] Danske Patienter: Program PRO . Anvendelse af PRO‐data i kvalitetsudviklingen af det danske sundhedsvæsen – anbefalinger og vidensgrundlag. [Program PRO. Use of PRO data in the quality development of the Danish healthcare system – recommendations and knowledge base.] In Danish. 2016. https://danskepatienter.dk/sites/danskepatienter.dk/files/media/Publikationer%20‐%20Egne/B_ViBIS/A_Rapporter%20og%20unders%C3%B8gelser/program_pro‐rapport.pdf (Accessed 06/10/2022)

[2] Nelson EC , Eftimovska E , Lind C , Hager A , Wasson JH , Lindblad S . Patient reported outcome measures in practice. BMJ. 2015;350:g7818. 25670183 10.1136/bmj.g7818

[3] Chen A , Väyrynen K , Schmidt A , et al. The impact of implementing patient‐reported measures in routine maternity care: a systematic review. Acta Obstet Gynecol Scand. 2022;101:1184‐1196. 36065150 10.1111/aogs.14446 PMC9812106

[4] Albinus N‐B . Ekspert i PROM: At udvikle spørgeskemaer til forskning er en disciplin isig selv. [Expert in PROM: Developing questionnaires for research is a discipline in itself]. In Danish. Dagens Medicin. 2021 apr. https://dagensmedicin.dk/ekspert‐i‐prom‐at‐udvikle‐spoergeskemaer‐til‐forskning‐er‐en‐disciplin‐i‐sig‐selv/ (Accessed 06/10/2022)

[5] Villumsen M , von Osmanski B , Lomborg K , Skov Benthien K . Klinisk brug af patientrapporterede oplysninger. [Clinical use of patient‐reported information – PRO]. In Danish. Frederiksberg 2022 https://www.frederiksberghospital.dk/ckff/pnw/Documents/Klinisk%20brug%20af%20patientrapporterede%20oplysninger%20‐%20PRO_tilg%C3%A6ngelig.pdf

[6] Nicolas‐Boluda A , Oppenheimer A , Bouaziz J , Fauconnier A . Patient‐reported outcome measures in endometriosis. J Clin Med. 2021;10:5106. 34768627 10.3390/jcm10215106 PMC8585017

